# Psoriasis localization patterns in the Swiss Psoriasis Registry (SDNTT) over 11 years: an analysis by sex and age

**DOI:** 10.1007/s00403-024-03375-5

**Published:** 2024-10-01

**Authors:** Ion Birkenmaier, Lara Valeska Maul, Iker Oyanguren, Christina Sorbe, Fabienne Fröhlich, Christoph Schlapbach, Kristine Heidemeyer, Nikhil Yawalkar, Wolf-Henning Boehncke, Hans-Christian Ring, Jacob P. Thyssen, Alexander Egeberg, Raphael Micheroli, Simon Francis Thomsen, Carlo Mainetti, Antonio Cozzio, Thomas M. Kündig, Mitchell P. Levesque, Alexander Navarini, Julia-Tatjana Maul

**Affiliations:** 1 Department of Dermatology, University Hospital Zurich, Gloriastrasse 31, 8091 Zurich, Switzerland; 2 Faculty of Science, University of Zürich, Zurich, Switzerland; 3 Department of Dermatology, University Hospital Basel, Basel, Switzerland; 4 Swiss4ward, Statistician and Data Analyst, Zurich, Switzerland; 5 Institute for Health Services Research in Dermatology and Nursing (IVDP), University Medical Center Hamburg-Eppendorf (UKE), Hamburg, Germany; 6 Department of Dermatology, Inselspital, University Hospital Bern, Bern, Switzerland; 7 Division of Dermatology and Venereology, Geneva University Hospitals, Geneva, Switzerland; 8 Department of Dermatology, Zealand University Hospital, Roskilde, Denmark; 9 Department of Dermatology, Bispebjerg Hospital, University of Copenhagen, Copenhagen, Denmark; 10 Department of Clinical Medicine, Faculty of Health and Medical Sciences, University of Copenhagen, Copenhagen, Denmark; 11 Department of Rheumatology, University Hospital Zurich, University of Zurich, Zurich, Switzerland; 12 Department of Biomedical Sciences, University of Copenhagen, Copenhagen, Denmark; 13 Department of Dermatology, Ente Ospedaliero Cantonale and Private Practice, Bellinzona, Switzerland; 14 Department of Dermatology, Cantonal Hospital St, Gallen, St. Gallen, Switzerland; 15 Faculty of Medicine, University of Zürich, Zurich, Switzerland; 16 Department of Biomedical Research, University of Basel, 4123 Allschwil, Switzerland

**Keywords:** Biologics, Psoriasis, Psoriasis treatment, Psoriasis area severity index, Localization, Registry data, SDNTT, Sex differences

## Abstract

Real-world data on anatomically localized psoriasis and its response to systemic therapy across different age-groups and sexes is limited. This study aimed to evaluate the severity and distribution of psoriasis over time in female and male patients receiving systemic therapies, categorized by age within the Swiss psoriasis registry (SDNTT). Patient-data was obtained over 11 years through the SDNTT. The localized Psoriasis Area and Severity Index (locPASI) of the head, trunk, upper and lower extremities was analyzed over two years following the start of systemic non-/biologic treatment. A total of 316 female and 517 male patients were analyzed. Male patients had a higher baseline locPASI for legs, trunk and arms (p < 0.001), but not for the head (p = 0.961). The locPASI for the head in younger female patients (18–40 years) had a higher score than those aged 55 + (p = 0.022) and after two years, middle aged (41–54) showed a lower score compared to younger patients (p = 0.045). Younger male patients revealed a lower score after two years of therapy in the leg- and arm-area compared to older (p = 0.018 and p = 0.048, respectively). Female patients on non-biologics had a fast initial response, converging with male patients’ scores over 24 months. Over 75% locPASI reduction was observed for female head-area (81.4%), male trunk (82.7%) and legs (76.1%). Absolute locPASI ≤ 2 was achieved 3–6 months for all locations with interleukin (IL)-17, IL-12/23 and IL-23-inhibitors, except for the legs of male patients on anti-IL-17 and female patients on anti-IL-12/23 and -IL-23. After two years, male patients did not achieve a locPASI ≤ 2 for any biologic-treatment in the legs, nor for the arms on anti-TNF-α. Significant disparities in localized PASI were observed between female and male patients. The age, sex and severity of distinct localizations should be considered to optimize treatment goals.

**Supplementary Information:**

The online version contains supplementary material available at 10.1007/s00403-024-03375-5.

## Introduction

Psoriasis is a heterogeneous inflammatory disease, affecting approximately 2-3% of the global population [1, 2]. It typically manifests as well-demarcated, scaly, erythematous plaques on various areas of the body. Depending on anatomical localization, these lesions greatly affect disease burden, and time to treatment response [3]. This can lead to substantial challenges in accurately assessing the severity of psoriasis and treatment strategies [4].

In addition, severity, treatment expectation and therapy response rate has been shown to vary between the sexes [5–7]. In a study analyzing the German and Swiss psoriasis registries PsoBEST and SDNTT, male patients had a higher disease severity in terms of the overall Psoriasis Area and Severity Index (PASI) at baseline. Female patients on the other hand had a higher response rate over one year of systemic treatment [6]. This was also corroborated in part by a Swedish registry study showing that female patients had a significantly lower PASI at enrolment in all measured areas, except for the head [5]. Such differences can affect the type of treatment patients receive [8], and greatly impact quality of life [9]. Furthermore, hormonal factors and age-related dynamics in psoriasis have been described for all life-stages [10]. These have been shown to not only impact patient needs and treatment goals [7], but also disease severity and therapy outcome [11–13].

Better understanding these intricacies and disparities between localization, treatment, age and the sexes over time can help guide clinical decision making and improve patient care [14, 15]. Despite this, long-term analysis of the treatment effectiveness of systemic therapies including IL-23 inhibitors on different body areas is still scarce. The aim of our study was therefore to provide valuable insights into the relationship between systemic therapy response, sex and age. We analyzed the localized (loc)PASI during two years of therapy, from patient data collected over 11 years in the Swiss psoriasis registry.

## Materials and methods

### Registry

The data for this study was retrieved from the SDNTT [6, 7, 16–25]. It includes informed and consenting, adult patients diagnosed with psoriasis, with or without psoriatic arthritis (PsA), who are receiving a systemic biological or non-biological therapy for the first time. As of June 2023, the following eight major university and municipal hospitals spread across each language region of Switzerland are participating to recruit patients [17]: University hospitals of Lausanne, Geneva, Bern, Basel and Zürich, and the cantonal hospitals of St. Gallen, Aarau and Bellinzona. The registry collects standardized data such as on the effectiveness and safety of dermatological biologics and systemic therapies for psoriasis, while adhering to the latest European guidelines [20, 26, 27]. Data are collected using paper- and web-based standardized case report forms and scheduled to be obtained in the clinic at predefined intervals following entry into the SDNTT: at baseline, 3-, 6- and 12-months and every six months thereafter up to 20 years per patient. All data is independently monitored and validated by the Centre of Excellence for Health Services Research in Dermatology at the University Medical Center Hamburg-Eppendorf, Germany.

### Patient data

This analysis includes patients enrolled in the registry between October 2011 and June 2023, with a minimum of one-year follow-up time after enrollment, over two-years of therapy. Patients receiving one of the following inclusion treatments were analyzed: Non-biologics (methotrexate, dimethyl fumarate, fumaric acid esters, ciclosporin, acitretin, apremilast), TNF-alpha-inhibitors (adalimumab, etanercept, infliximab, certolizumab, golimumab, incl. biosimilars), IL-17-inhibitors (secukinumab, ixekizumab), IL-12/23 and IL-23 inhibitors (ustekinumab, guselkumab, tildrakizumab, risankizumab) collectively called IL-23 inhibitors hence-forth. Patients were sub-grouped by sex (male vs. female), age reflective of hormonal life-cycles (18–40, 41–54 and 55+) and by inclusion treatment group. Patients with more than one or without inclusion treatment validated by follow-up-information were excluded. Variations in visiting schedule was accounted for by allowing for +/ – 1 month during the first two visits after 3 and 6 months and +/ – 2 months for visits at months 12, 18 and 24.

Following the registry protocol, patients are allowed to stop or switch treatment at any point in time. Patients with such treatment discontinuations were only taken into analyses until treatment was stopped, e.g. patients stopping adalimumab after 19 months were included at month 0, 3, 6, 12 and 18, but excluded for month 24.

### Variables

Severity of psoriasis was assessed using the PASI scoring system as described by Fredriksson and Pettersson [28]. To discern the severity of psoriasis within individual body areas, we calculated a localized PASI score, termed locPASI [29]. Unlike the standard PASI scoring system, locPASI calculation did not include the surface area multiplier in order to focus on the severity of psoriasis in each specific body regions, irrespective of their relative size. Thus, the locPASI also ranges from 0 to 72, but for each localization individually.

In addition, patient information on age, sex, nail involvement, Dermatology life Quality Index (DLQI), PsA, Body Mass Index (BMI), smoking status and disease duration were considered for these analyses.

### Statistical analysis

The descriptive analyses for each of the variables were performed using standard descriptive statistics (relative frequencies for the qualitative variables, and the mean together with the interquartile range for the quantitative variables). To do the comparison at baseline between each group, the Mann-Whitney-Wilcoxon [30, 31] test was used for the quantitative variables and Fisher exact probability test [32] for the qualitative variables.

Patient subgroups allowed development of individual models for each part of the body. In each model the locPASI served as the dependent variable, while age (continuous), sex (dichotomous), nail involvement (dichotomous), DLQI (continuous), PsA (dichotomous), BMI (continuous), smoking status (dichotomous) and disease duration (continuous) acted as independent variables.

Patients who had missing values in one or more body area or in Body Surface Area were not included in the analysis (as-observed-analysis).

For comparisons after baseline, the Shapiro-Wilks [33] test was used for normality and the t test [34], establishing an alpha of 0.05 for all cases. All analyses were performed with R (version 4.1.1.) and RStudio [35, 36].

## Results

### Patient demographics and baseline characteristics

**Fig. 1:**
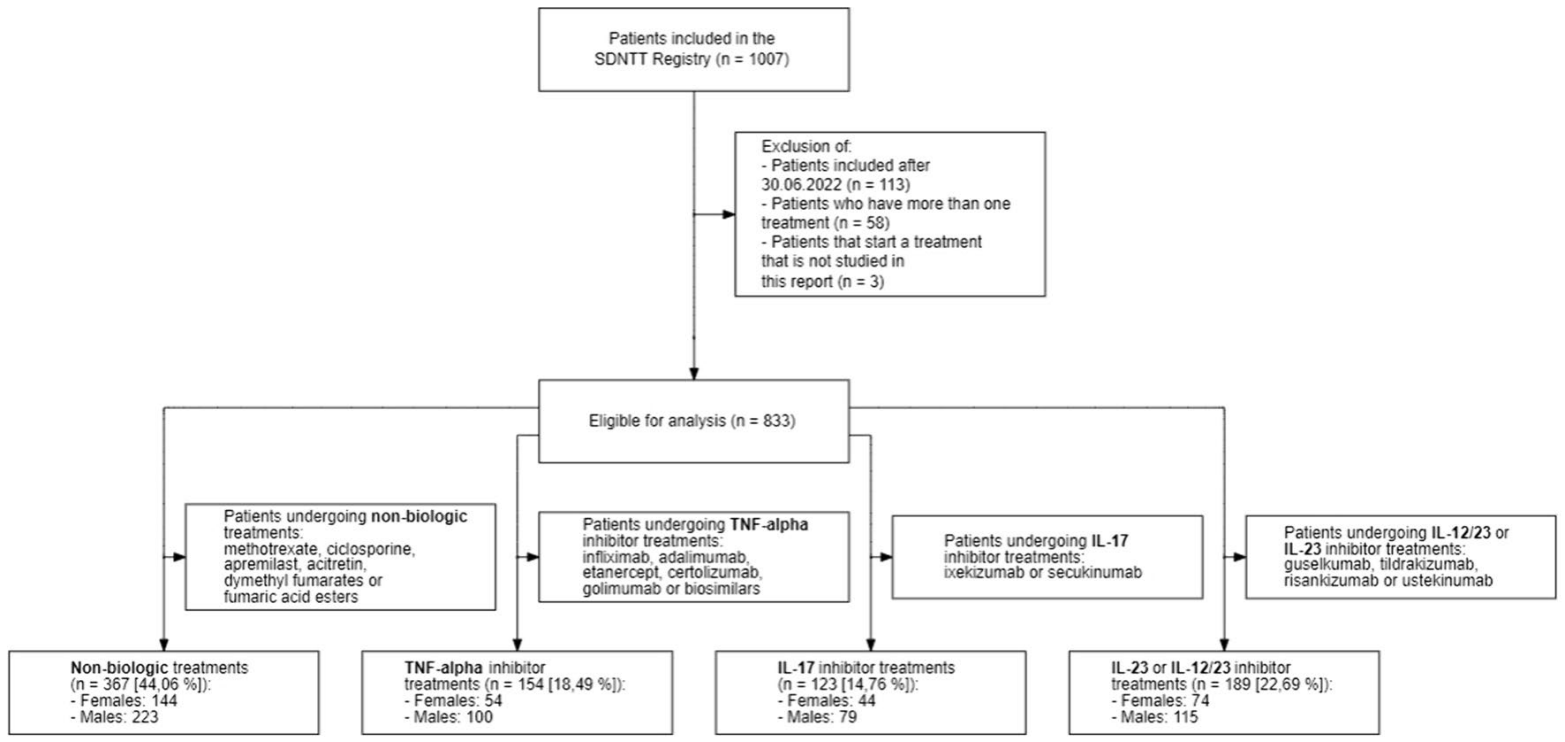
Flowchart of patients eligible for analysis. A total of 1007 patients were registered in the SDNTT as of June 2023. 174 patients were excluded due to time since entry into the registry, number of therapies, or type of therapies received. 833 patients were segmented into four treatment groups for analysis, reflecting patients having received non-biologic, anti-TNF-alpha, anti-IL-17 and anti-IL23 or IL-12/23 therapies. In total, 367 patients received non-biologic treatments (144 females and 223 males) and 466 biologic therapies (172 females and 294 males)

Of the 833 patients eligible for analysis (Fig. 1), 62% were male (n = 517) and 38% female (n = 316). Overall, male patients had significantly higher median PASI and BMI at baseline than female patients (p < 0.001), but there were no differences in age, smoking status, disease duration and diagnosis of PsA (p > 0.05) (Table 1). There was no difference in the relative number of patients receiving biologic therapies (p = 0.288; Supplementary Table SI and SII).

**Table 1:** Main patient characteristics, demographics and clinical information at baseline

	All patients (n = 833)				
	No. of evaluable male patients	male (n = 571)	No. of evaluable female patients	female (n = 350)	*p* value
Median PASI (IQR)	507	8.9 (5.6–12.6)^a,*^	310	7.0 (3.7–11.2)^a,*^	< 0.001
Median Age in years (IQR)	516	45.0 (36.0–54.0)^a^	316	44.0 (31.0–56.0)^a^	0.139
Median BMI (IQR)	492	27.1 (24.1–30.6)^a,*^	296	25.0 (21.8–29.7)^a,*^	< 0.001
Median Disease duration (years)	430	16.2 (6.0–24.0)^a^	257	16.1 (5.0–23.0)^a^	0.526
Ongoing PsA		20%^b^		18%^b^	0.326
Current smokers		43%^b^		44%^b^	0.910

*PASI* Psoriasis Area Severity Index, *IQR* InterQuartile Range, *BMI* Body Mass Index; kg/m^2^, *PsA* Psoriatic Arthritis

^a^Wilcoxon test, ^b^Fisher exact test

*p < 0.001

Median PASI and BMI showed significant differences between the analyzed female and male patient groups. There were no differences in median age, disease duration, PsA and smoking status

### Sex distribution of localized PASI

**Fig. 2:**
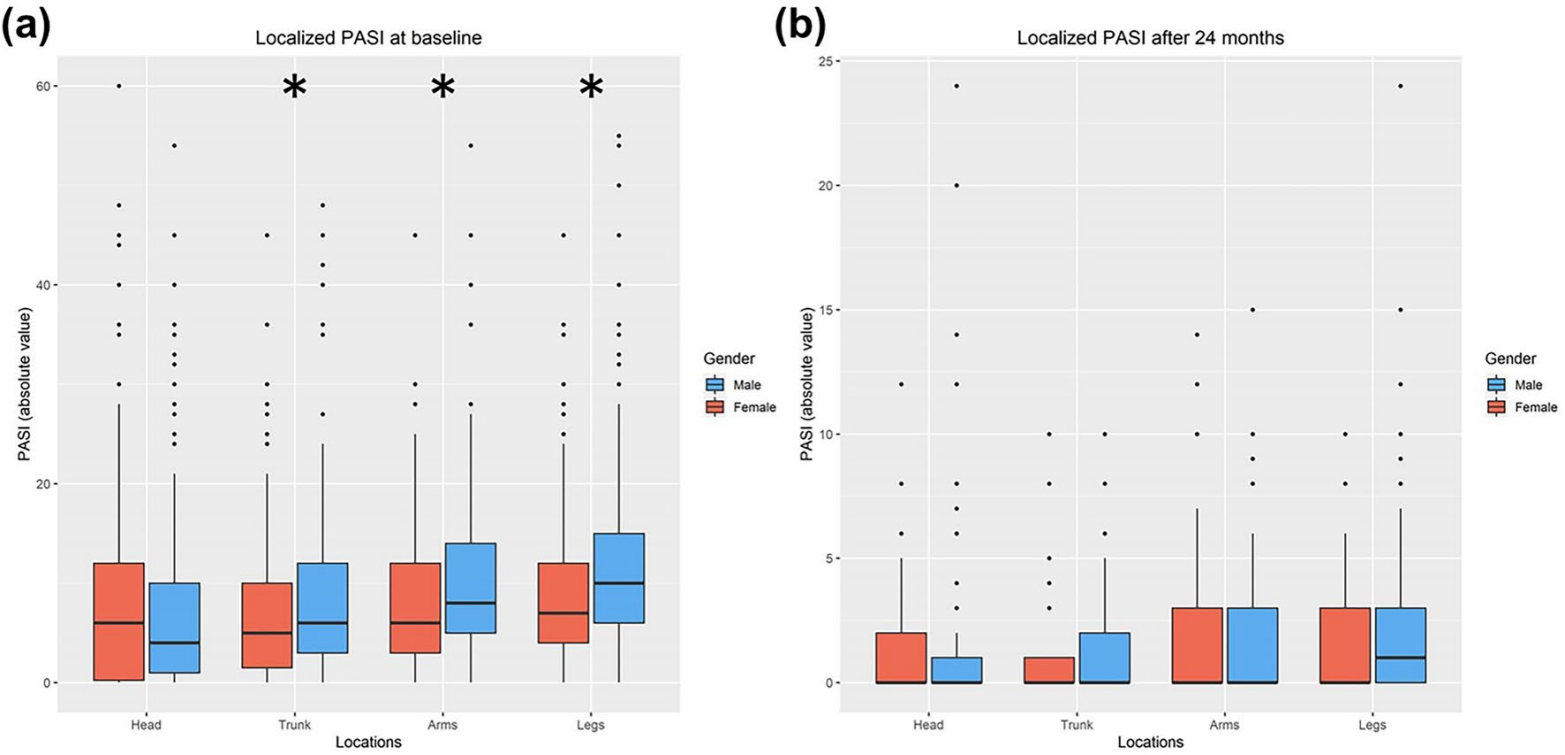
**a–b** Localized PASI of female and male patients treated with any systemic therapy. At baseline (**a**), Trunk, arms and legs were significantly higher in male compared to female patients (*p ≤ 0.001). After two years of treatment (**b**), there were no significant differences. PASI (Psoriasis Area and Severity Index)

The locPASI for head, trunk, arms, and legs was compared between female and male patients at baseline and after two years of treatment for all patients independent of treatment (Fig. 2). Male patients had a higher locPASI (standard deviation [SD]) for legs (11.30 [SD8.48] vs. 9.21 [SD7.72]), trunk (8.17 [SD7.25] vs. 7.14 [SD7.64]) and arms (9.78 [SD6.96] vs. 7.76 [SD6.63]) (all p < 0.001), but not for the head (7.27 [SD8.49] vs. 9.21 [SD10.79]) (p = 0.961) (p < 0.001) (Fig. 2a). After two years follow-up, there were no significant differences for any locations (Fig. 2b).

### Differences in localized PASI between age groups

Female and male patients between 18 and 40 years of age (younger) were compared to patients 41–54 (middle aged) and 55+ (older) at baseline and after two years of follow-up (Supplementary Table SIII). While there were no differences at baseline between young female patients compared to middle aged, young patients had a higher locPASI for the head than older patients (10.22 [SD10.81] vs. 7.96 [SD11.10]; p = 0.022). After two years of therapy, the head-area was no longer significantly different between young and older female patients, but middle aged now had a lower locPASI score compared to younger patients (0.71 [SD1.71] vs. 1.94 [SD3.01]; p = 0.045). All other areas were comparable at baseline and after two years of therapy.

In male patients, there were no differences at baseline, except for a lower locPASI of the head in the middle-aged group compared to younger patients (6.42 [SD7.8] vs. 8.09 [SD8.83]; p = 0 0.033). After two years of therapy, the leg and arm-area showed a significantly lower locPASI in the younger compared to the older group (1.61 [SD2.25] vs. 3.04 [SD3.33]; p = 0.018 and 1.26 [SD1.89] vs. 2.16 [SD2.54]; p = 0.048 respectively).

### Localized PASI by therapy between female and male patients

#### Non-biologic therapies

**Fig. 3:**
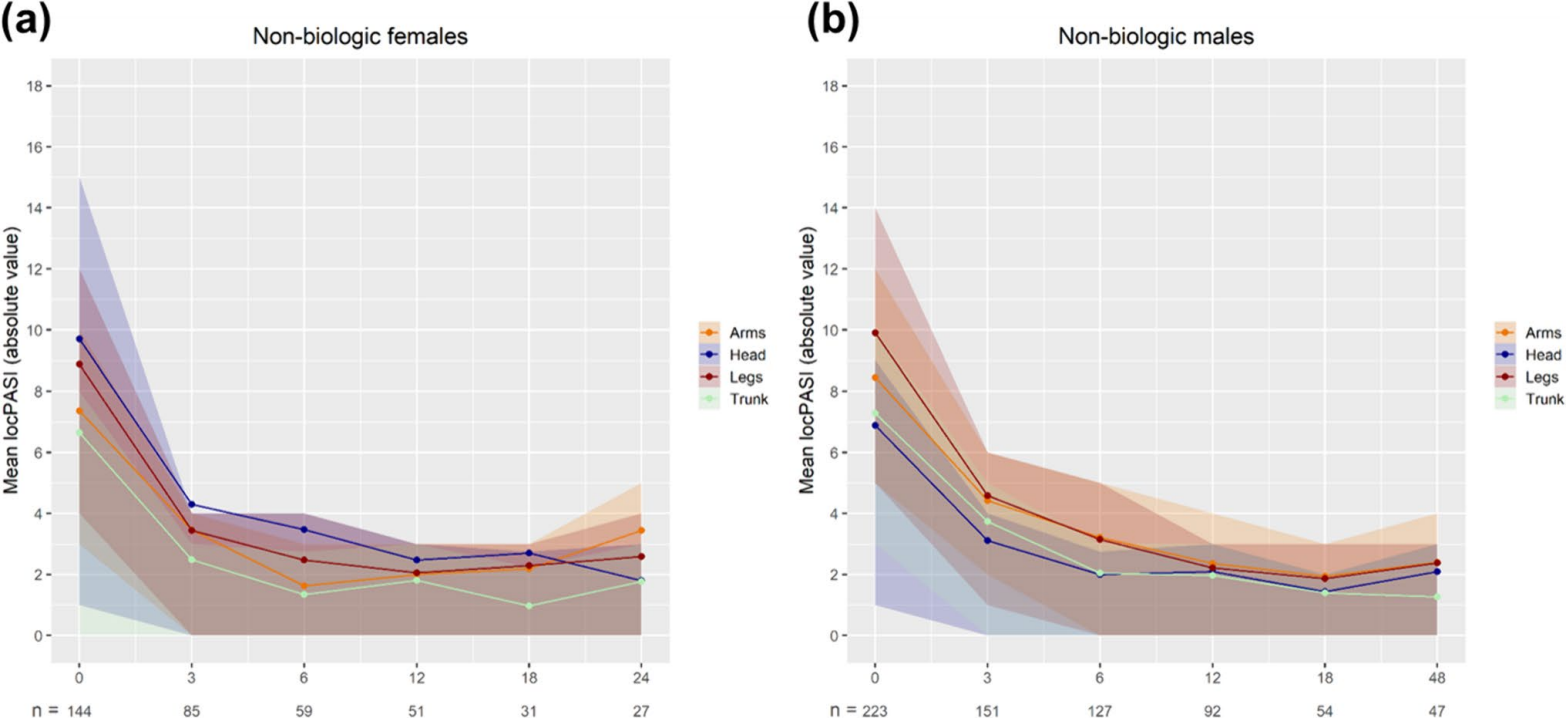
**a-b** Mean locPASI of the head, trunk, arms and legs over two years of systemic non-biologic treatment (methotrexate, fumarates, retinoids, cyclosporine and apremilast). The two graphs show progression of locPASI for female (**a**) and male patients (**b**). The colored areas around the curves represent the confidence intervals. Please note that the time intervals on the X-axis are not equally spaced and decreasing number of patients due to treatment discontinuations. locPASI (Localized Psoriasis Area and Severity Index)

In terms of absolute locPASI, male patients receiving non-biologic systemic treatments had a higher score at baseline for all areas (p < 0.05) except the head (p = 0.256) compared to female patients (Fig. 3). Male patients still had a significantly higher locPASI for the arms and trunk after three months of treatment, but not for the head and legs. After two years of therapy, there were no significant differences in locPASI between the sexes for any location (Supplementary Table SIV).

After three months of therapy, female patients had an average locPASI reduction of 58.2%, compared to 51.2% in male patients (absolute locPASI reduction of 4.73 vs. 4.17 respectively), while after two years male patients had a reduction of 75.0% compared to 69.8% in female patients (absolute locPASI reduction of 6.10 vs. 5.75 respectively). A relative locPASI reduction of more than 75% was only achieved for female head-area (81.4%), as well as male trunk (82.7%) and legs (76.1%) (Supplementary Table SIV).

#### Biologic therapies

**Fig. 4:**
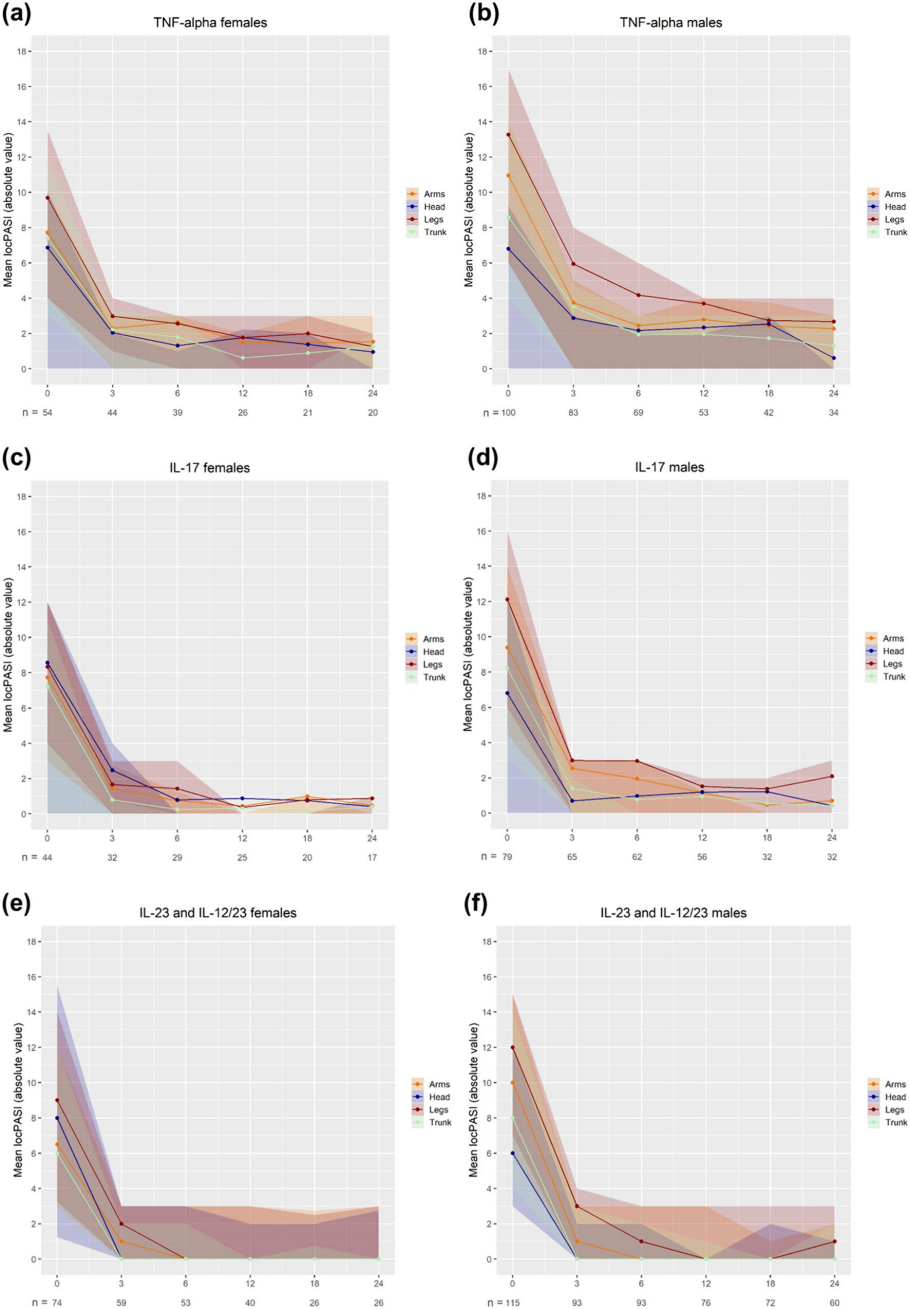
**a–f** Mean locPASI of the head, trunk, arms and legs over two years of biologic treatment. Individual graphs show the progression of locPASI over time for female (**a**, **c**, **e**) and male patients (**b**, **d**, **f**) treated with (**a**, **b**) TNF-α inhibitors (adalimumab, certolizumab, etanercept, golimumab, infliximab), (**c**, **d**) IL-17 inhibitors (secukinumab and ixekizumab) and (**e**, **f**) IL-23 and IL-12/23 inhibitors (ustekinumab, tildrakizumab, risankizumab and guselkumab). The colored areas around the curves represent the confidence intervals. Please note that the time intervals on the x-axis are not equally spaced and decreasing number of patients due to treatment discontinuations. *PASI* (Psoriasis Area and Severity Index)

Over all areas, biologic therapies showed a strong response during the two-year observation period (Fig. 4). In particular, most patients receiving IL-17 and IL-23 inhibitors achieved a mean locPASI≤2 for all localizations within the first 3-6 months of therapy (Fig. 4c–f). While TNF-α inhibitors were not as consistently fast, after two years of therapy, female patients achieved a mean locPASI≤2 in all localizations (Fig. 4a). However, some male patients did not, specifically in the leg-area under any biologic therapy and the arms under TNF-α inhibitors (Supplementary Table SV).

Comparing the locPASI at baseline, male patients receiving either TNF-α and IL-23 inhibitors had a significantly higher score for the arms than female patients (p≤0.003), while male patients receiving TNF-α and IL-17 inhibitors had a higher score for the legs (p≤0.028). Nevertheless, after 24 months, there were no differences in locPASI between female and male patients for each of the biologic treatment groups (Supplementary Table SV).

## Discussion

This study analyzed the differences in localized psoriasis severity over two years of treatment with biologic and non-biologic therapies. This is the first study further stratifying results by sex and age over such a long treatment period and including more recent biologics. Our data suggest that there are baseline and short-term differences between the sexes and age groups that converge over time, but that should be considered in clinical decision-making. For example, our data support the concomitant use of topical therapies to help reduce the burden of disease, especially in the initial stages of starting a new systemic therapy or during flares [37]. As the localization of psoriatic plaques not only influences quality of life [4, 38], but also treatment effectiveness [3, 39], these results provide an important foundation for personalized patient therapy.

### Baseline severity and age

Gender, lesion distribution, and disease severity play pivotal roles in determining the choice between non-/biologic therapies [14]. Our analysis of the baseline locPASI between male and female patients revealed that, with the exception of the head, male patients had a higher score for all localizations. Despite this, both sexes received the same relative number of non-biologic and biologic therapies. In contrast to previous studies reporting higher PASI-scores in males and resulting disparities in treatment patterns [8, 40, 41], our findings are more in line with recent research demonstrating a balanced approach to prescription practice [6, 42, 43]. This may be explained by the availability and increased safety-profile of more recent biologics [44], as well as the diligent updates of the European guidelines [26, 27] that are continuously adopted in individual countries [45–47].

Interestingly, our observation of baseline difference in the head-area between the sexes, aligns with recent findings from a Swedish registry study [5]. They speculated that hair length and care may be two of the main drivers for the higher PASI in the head localization of female patients. Building on this, we have uncovered an interesting baseline disparity in locPASI based on age, showing a higher score in the head-area of younger (18–40 years) compared to older (55+) female patients. This discrepancy prompts further exploration into the contributing factors, beyond those proposed in the Swedish study. One notable aspect we consider is the potential influence of hormonal life-cycles on psoriasis severity. Research has documented the impact of sex-hormones on the pathophysiology of psoriasis10, with studies indicating their role in modulating not only disease severity but also factors relevant to hair growth, sebaceous-gland function [48], and keratinocyte activity [49]. These intricate interplays may offer insights into the observed age-dependent variations in baseline psoriasis severity among female patients. Furthermore, while our data indicate no baseline differences in psoriasis severity for male patients across age groups, we discovered a trend among male patients revealing age-related variations in locPASI for the arms and legs after two years of therapy. Specifically, older male patients exhibited a significantly higher locPASI compared to their younger counterparts.

These age- and sex-dependent differences suggest that the disease may manifest and respond differently to treatment in various age-brackets among the sexes, underscoring the importance of considering such factors in the assessment and management of psoriasis.

### Non-biologic therapies

Our data on non-biologic therapies could show that while female patients may respond fast initially, their long-term response remains largely consistent. Male patients on the other hand appear to have a slightly slower response, but continue to improve over time. After two years, we could not show a significant difference for the locPASI in any of the localizations between the sexes. While previous publications have observed that some localization may respond differently to therapy, these focused primarily on special areas, such as palms-, scalp- or genital-area [3, 4], or shorter treatment periods [6]. Our data complement and extend these findings by considering a broader area of localizations over a two-year treatment period. This suggests that while there are differences in the short-term treatment course, these may converge over an extended period. As managing patient expectations can have significant impact on treatment adherence [50], it is important to consider such changes over time. In particular, considering the clinical implication of sex differences as observed here can help tailor treatment plans to individual patient needs, such as prescribing a topical treatment to bridge the time until response.

### Biologic therapies

Our analysis of biologic-therapies demonstrate a robust and sustained response over two-years. Notably, the efficacy of IL-17 and IL-23 inhibitors was high, with a majority of patients achieving a locPASI≤2 across most localizations within the initial 3-6 months of therapy. Overall, female patients achieved a locPASI≤2 in all localizations after two years of any biologic treatment. However, there were notable variations among male patients. Particularly the leg-area for all treatments and the arms under TNF-α inhibitors did not reach a locPASI≤2 at any time point, suggesting the leg-area to be a potentially harder to treat location, with differences in treatment response between the sexes.

On the other hand, our longitudinal analysis demonstrated a convergence in locPASI scores between female and male patients across all biologic treatment groups by the end of the 24-month observation period. This was observed despite male patients exhibiting higher baseline scores for the arms under TNF-α and IL-23 inhibitors and for the legs under TNF-α and IL-17 inhibitors.

The observed differences in baseline scores and the subsequent convergence in locPASI suggest that while initial responses may vary, similarly to non-biologic therapies, the long-term effectiveness of biologic therapies equalizes between male and female patients. This finding further underscores the importance of considering not only short-term outcomes but also the sustained efficacy of treatments over extended periods, while also enforcing the benefit of biologics for both sexes.

### Strengths and Limitations

The strengths of this study lie in its comprehensive analysis of non-biologic and biologic therapies over a two-year period, utilizing a diverse dataset that includes longitudinal follow-up information from over 11 years of data collection. The inclusion of such diverse treatment modalities enhances the resolution of the findings, providing a holistic view of psoriasis therapy outcomes. The long observation time allows for a nuanced understanding of the evolving dynamics of treatment response, shedding light on both short-term variations and long-term trends. Moreover, the incorporation of age and sex as factors revealed significant differences in psoriasis severity and treatment response.

However, it is crucial to acknowledge the limitations inherent in registry data, including potential biases and declining number of patients over time. Thus, the grouping of all non-biologic therapies, despite differences in their mechanisms of action, as well as the anti-IL-12/23 and -IL-23 biologics, was a necessary compromise to address the challenge of low patient numbers. Another notable limitation is the calculation of the localized PASI score without incorporating the surface area multiplier (locPASI). While this provides a more individualized assessment of PASI-scores for each localization, it may impact the comparability of results with studies using the conventional PASI calculation. Furthermore, treatment discontinuations and following exclusion from further analyses, may show a “healthy survivor effect”, since especially patients with safety issues or insufficient treatment response are those to stop treatment. Finally, the concomitant “on-demand” use of topical treatments with systemic therapy was not analyzed. While confounding is unlikely due to the efficacy of systemics, it cannot be ruled out.

Despite these limitations, the utilization of registry data offers a real-world perspective, reflecting the diversity of patient populations encountered in clinical practice, and contributes valuable information to the ongoing discourse on personalized approaches to psoriasis management.

## Conclusion

In conclusion, our study emphasizes the role of psoriatic plaque localization in tailoring effective treatment strategies. A comprehensive analysis of this scale has not been done before. As such, we observed sex-differences in baseline disease severity for different localizations, potentially stronger early treatment response in female patients, and converging of response over a two-year period. This highlights the dynamic nature of psoriasis management, as the observed distinctions can greatly impact adherence, treatment satisfaction and ultimately patient well-being. Furthermore, the age-related variations identified in our research underscore the potential influence of hormonal life cycles on treatment outcomes, warranting a more detailed exploration into these intricate dynamics. Importantly, our findings stress the significance of considering sex and age in both short-term and long-term outcomes when formulating individualized treatment plans. These are crucial for developing more effective and nuanced approaches that account for the diverse needs of psoriasis patients, ultimately improving their overall and long-term quality of life.

### Use of AI or language model (LLM)

Basic tools were used to improve vocabulary and grammar. No AI, language model, machine learning, or similar technologies were used in the creation or editing of any of the content in this submission. All content is unique and original.

## Key points

- ***Why was the study undertaken?***
- Psoriasis is a debilitating skin disease that appears to cause stronger symptoms in male patients.
- Female patients can have better treatment outcomes with systemic therapy.
- It is not clear how long-term systemic treatment effects psoriasis severity in each body area individually and whether there are differences between the sexes.
- ***What does this study add?***
- There are distinct age-related differences in psoriasis severity. At baseline, young female patients have a significantly higher PASI for the head compared to older female patients.
- Female patients appear to respond faster to treatments within the first months, but converge with male patients after two years.
- ***What are the implications of this study for disease understanding and/or clinical care?***
- Patients should be treated based on their individual needs.
- Age plays an important role in the clinical symptoms and potentially treatment effectiveness.
- Female patients may respond faster, but male patients catch up over time, thus treatments should be given enough time to reach their full effect, while considering individual patients’ needs and goals.

## Supplementary Information

Below is the link to the electronic supplementary material.

Supplementary file1 (XLSX 10 KB) Supplementary Table SI. Absolute and relative number of patients treated with non-biologic and biologic therapies

Supplementary file2 (XLSX 14 KB) Supplementary Table SII. Descriptive and comparative data tables per visit and localization for all female and male patients independent of treatment

Supplementary file3 (XLSX 34 KB) Supplementary Table SIII. Descriptive and comparative data tables per visit and localization for all female and male patients of different age groups (18-42, 51-54, 55+) treated with non-biologic and biologic therapies

Supplementary file4 (XLSX 26 KB) Supplementary Table SIV. Descriptive and comparative data tables per visit and localization for female and male patients treated with non-biologic therapies

Supplementary file5 (XLSX 61 KB) Supplementary Table SV. Descriptive and comparative data tables per visit and localization for female and male patients treated with biologic therapies

## Data Availability

Due to the nature of the medical information utilized in this manuscript, which includes registry data containing patient-specific details, the raw data cannot be made available publicly. The restrictions are in accordance with Swiss privacy laws and the terms of the informed consent agreements signed by the participants. Therefore, access to the data is limited to ensure the confidentiality and privacy of the patient information. Any requests for further details about the analysis, the dataset and its use can be directed to the corresponding author, subject to compliance with the relevant privacy regulations and upon reasonable request.

## Declarations

### Use of AI or language model (LLM)

Basic tools were used to improve vocabulary and grammar. No AI, language model, machine learning, or similar technologies were used in the creation or editing of any of the content in this submission. All content is unique and original.
